# Prostate tumor overexpressed-1, in conjunction with human papillomavirus status, predicts outcome in early-stage human laryngeal squamous cell carcinoma

**DOI:** 10.18632/oncotarget.8103

**Published:** 2016-03-15

**Authors:** Lin Yang, Hongzhi Wang, Yan Wang, Zhenyu He, Haiyang Chen, Shaobo Liang, Shasha He, Shu Wu, Libing Song, Yong Chen

**Affiliations:** ^1^ Sun Yat-Sen University Cancer Center, Guangzhou, China; ^2^ State Key Laboratory of Oncology in Southern China, Guangzhou, China; ^3^ Collaborative Innovation Center for Cancer Medicine, Guangzhou, China; ^4^ The Six Affiliated Hospital of Sun Yat-sen University, Guangzhou, China; ^5^ The First Hospital of Foshan, Foshan, China

**Keywords:** prostate tumor overexpressed-1, laryngeal squamous cell carcinoma, prognosis, HPV, biomarker

## Abstract

In human cancer, molecular markers combined with clinical characteristics are used increasingly to predict prognosis. Prostate tumor overexpressed-1 (PTOV1), first identified in prostate cancer, is a key factor in tumor progression and correlates with unfavorable clinical outcomes. HPV infection status was tested by HPV E6-targeted multiplex real-time PCR and p16 immunohistochemistry (IHC). Real-time PCR and western blotting analyses were used to examine the mRNA and protein expression levels of PTOV1 in eight paired LSCC samples. IHC was performed to assess PTOV1 protein expression in 196 paraffin-embedded, archived LSCC samples. PTOV1 protein and mRNA expression was increased in LSCC tissues compared with adjacent noncancerous tissue samples. High expression of PTOV1was significantly associated with advanced TNM stage by the X^2^ test. Multivariate analysis revealed that PTOV1 and HPV status were independent prognostic indicators of overall survival (OS) and progression-free survival (PFS) (*P* = 0.001, *P* = 0.009 for OS, *P* = 0.005, *P* = 0.012 for PFS, respectively). Our study provides the first evidence that the combination of PTOV1 expression level and HPV status provides more prognostic information compared with HPV status alone with the significance still exists in the HPV negative subgroup.

## INTRODUCTION

Squamous cell carcinoma (SCC) of the head and neck represents more than 90% of all head and neck cancers, and ranks as the sixth most common cancer worldwide [1]. With an annual estimated 159,000 new cases and 90,000 related deaths, laryngeal carcinoma is largest contributor to head and neck squamous cell carcinoma (HNSCC) [2]. Human papillomavirus (HPV), especially type 16, is emerging as an important factor in developed countries, and HPV-induced HNSCC has distinct epidemiology and biology, especially a more favorable prognosis, compared with the HPV negative HNSCC [3, 4].

In clinical practice, treatment decision-making in LSCC is mostly based on the TNM staging system in the absence of a universal prognostic staging system to predict clinical outcomes for patients [5]. However, in addition to the traditional clinical staging system and pathological standards, novel loss of function alterations of the alterations of the EGFR mutation, overexpression gene P53 and activation of oxidative stress factor NFE2L2 also found in laryngeal tumors and have prognostic value for clinical outcome [6-8]. However, none of these biomarkers has considered the HPV status, which is also an important determinant of survival outcome.

The prostate tumor overexpressed-1 (PTOV1) gene has 12 exons and is located on a region of chromosome 19 (19q13) that is amplified in HNSCC [9]. *PTOV1* was first identified in prostate cancer during a differentially screening for genes expression [10]. Additionally, PTOV1 is involved in the development and progression of human malignancies, such as lung, endometrium, bladder, kidney and ovary cancer [11]. However, little is known about the expression and clinical significance of PTOV1 in LSCC.

Ectopic expression of PTOV1 promoted entry into the S phase of the cell division cycle to promote mitotic activity [12]. High levels of PTOV1 in prostatic tumors correlate with the Ki67 proliferative index, indicating that increased PTOV1 expression is functionally related to the cells' proliferative status [11]. Marques *et al*. reported that PTOV1 enhanced the expression of c-Jun protein at the post-transcriptional level, and is required for cell invasiveness and motility [13]. A recent study demonstrated that the PTOV1 is thought to be a vital determinant in cancer treatment with retinoids by repressing retinoic acid receptor (RAR) activity [14].

In this study, we assessed the expression of PTOV1 in a series of LSCC specimens and investigated its associations with clinicopathological parameters and prognosis in patients with LSCC survival.

## RESULTS

### HPV status and type distribution

16.8% (33/196) of the tumors were HPV-positive (HPV DNA-positive/p16-overexpression), among which 72.7% (24/33) were HPV type 16. The other HPV types include 18 (one tumor), 35 (one tumor), 45(three tumor), 56 and 81 (two and three tumors, respectively). One tumor positive for HPV 16 simultaneously included a second HPV type (18).

### PI3K mutation status

The overall rate of PI3K mutation was 1.53% (3/196), which all involved exons 9. Two of the three mutation site was codon 545(codon 1634 A > C, protein 545 E > A; codon 1634 A > G, protein E > G). The other mutation site was codon 542(codon 1624 G > A, protein E > K;).

### PTOV1 is overexpressed in human LSCC tissues and is associated with LSCC progression

**Figure 1:**
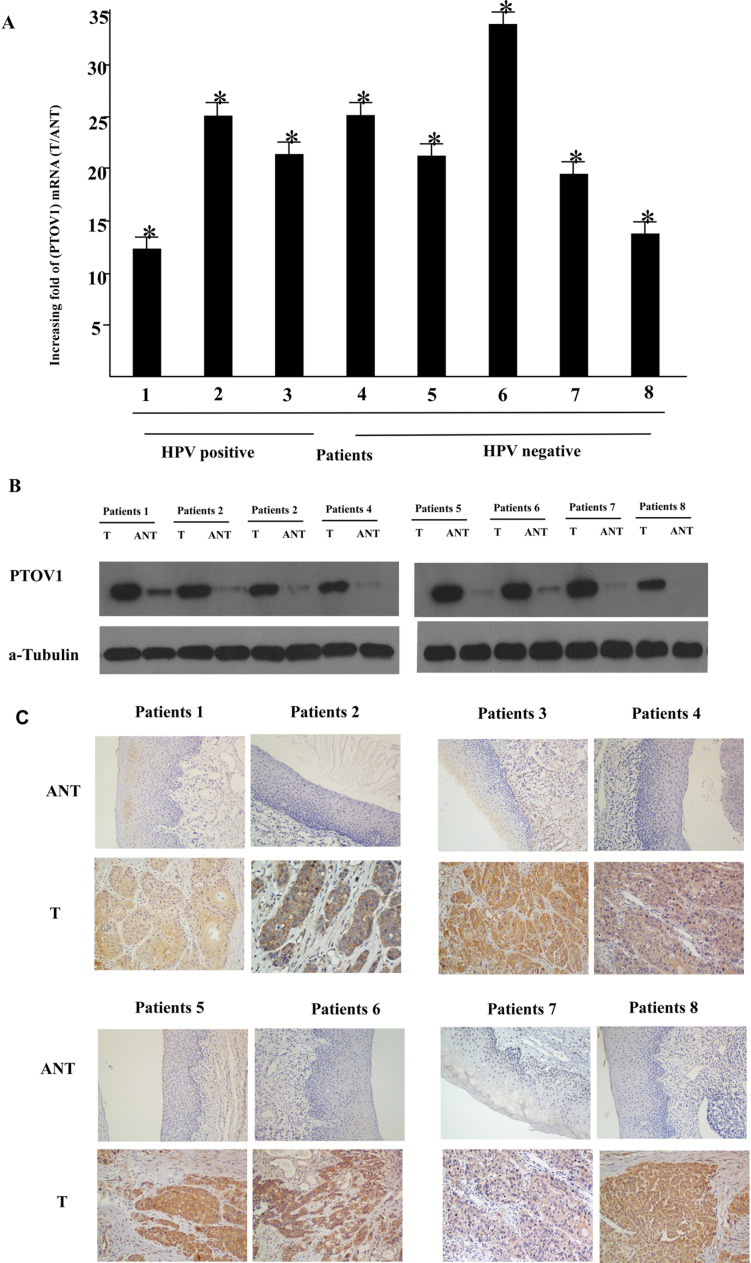
Overexpression of PTOV1 mRNA and protein in human laryngeal squamous cell carcinoma (LSCC) tissues **A.** PTOV1 mRNA expression in eight matched pairs of LSCC tissues (T) and adjacent non-cancerous tissues (ANT), as quantified by PCR and normalized to the expression of *GAPDH*. Error bars are the standard deviation of the mean (SD) for three experiments performed in parallel. **B.** Representative western blotting analyses of PTOV1 protein expression in eight pairs of matched LSCC tissues; α-tubulin was used as the loading control. **C.** Immunohistochemical analysis of PTOV1 protein expression in the eight pairs of matched LSCC tissues, *P < 0.05.

To determine whether PTOV1 is overexpressed in human LSCC, eight paired tumor samples (T) and the adjacent non-cancerous tissues (ANT) from the same patients were subjected to RT-PCR and western blotting analyses. As shown in Figure 1A, PTOV1 mRNA was expressed at significantly higher levels in the LSCC tissue samples than in the non-cancerous tissues, with at least 10-fold higher levels observed in the tumor tissues. Consistent with the mRNA analysis, PTOV1 protein levels were also increased in LSCC tissues compared with the surrounding non-tumor regions (Figure 1B).

PTOV1 was mainly localized in the tumor cell cytoplasm, with strong nuclei staining occasionally observed; little or no expression of PTOV1 was observed in the normal epithelial cells (Figure 1C). The positivity rates for PTOV1 were 94.5%. Using cutoff scores of ≤ 4 (low) and ≥ 6 (high), 119/196 (60.7%) cases were classified as high PTOV1-expressing and 77/196 (39.3%) as low PTOV1-expressing.

**Figure 2:**
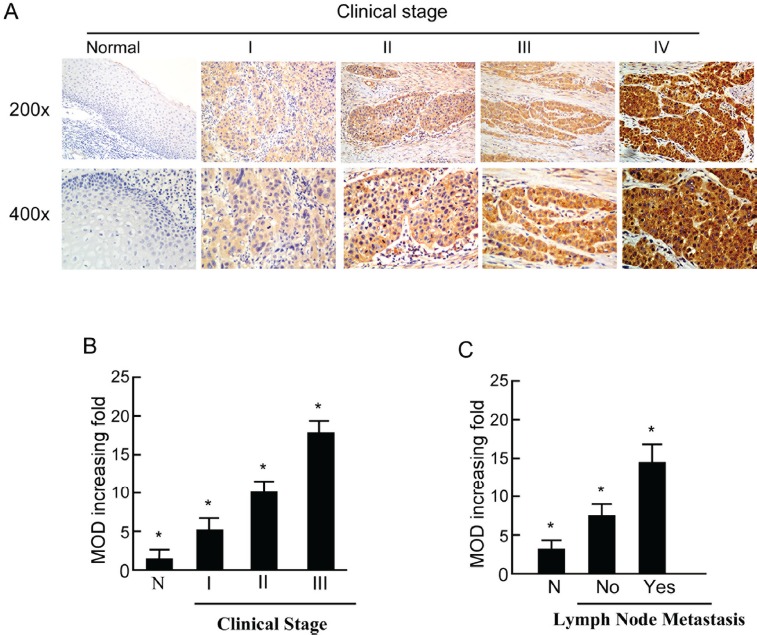
Expression of PTOV1 in different clinical stages of laryngeal squamous cell carcinoma (LSCC) **A.** Representative images of immunohistochemical staining for PTOV1 in normal (control sections) LSCC tissues and different clinical stages of LSCC. **B.** Average fold-change in the mean optical density (MOD) for PTOV1 in different clinical stages of LSCC compared with normal laryngeal tissues. **C.** The statistical analyses of the average MOD of PTOV1 staining in the lymph node metastasis group and the lymph node metastasis-free group, *P < 0.05.

**Figure 3:**
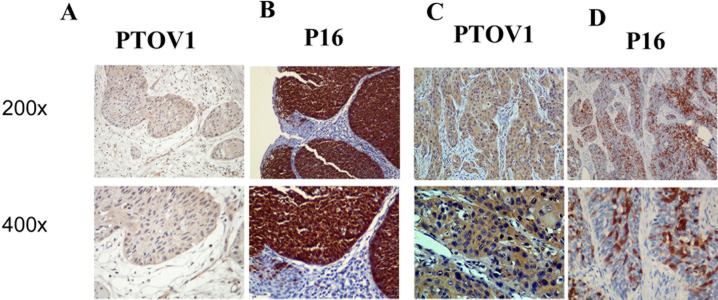
Same region of same tumor staining positive for PTOV1 (A, C; 200×, 400×) and p16 (B, D; 200×, 400×)

Furthermore, PTOV1 expression in the LSCC increased with increasing clinical stage, as shown by IHC staining intensity (Figure 2A). Quantitative IHC analysis revealed that the mean optical density (MOD) values of PTOV1 staining in all LSCC samples were higher than those in the normal control laryngeal tissues. Additionally, the MOD values of PTOV1 staining significantly increased with progression from stage I to III (*P* < 0.05, Figure 2B). Moreover, the MOD values of PTOV1 staining were markedly higher in the lymph node metastasis group than in the lymph node metastasis-free group (*P* < 0.001, Figure 2C). Figure 3 shows a tumor positive for both PTOV1 and p16 (as a surrogate marker for HPV) [15].

We further analyzed the association between PTOV1 and the clinicopathological characteristics of LSCC using the χ^2^ test. Compared with patients with low PTOV1-expressing tumors, patients with high PTOV1-expressing tumors tended to with the characteristics of being male (*P* = 0.005), in advanced T stage (*P* = 0.002), advanced N stage (*P* = 0.007), advanced stage (*P* = 0.001), showed disease progression (*P* = 0.019) and had poorer survival (*P* < 0.001) (Table 1).

**Table 1:** Association between PTOV1 expression and the clinicopathological features of the LSCC patients

Features	Total	Low expression (%)	High expression (%)	*P*
**Age (years)**				0.310
< 60	103 (52.6%)	37 (35.9%)	66 (64.1%)	
≥ 60	93 (47.4%)	40 (43.0%)	53 (57.0%)	
**Gender**				0.005
Male	181 (92.3%)	66 (36.5.0%)	115 (63.5%)	
Female	15 (7.7%)	11 (73.3%)	4 (26.7%)	
**Drinking status**				0.803
Absent	29 (14.8%)	12 (41.4%)	17 (58.6%)	
Present	167 (85.2%)	65 (38.9%)	102 (61.1%)	
**Smoking status**				0.139
Absent	112 (57.1%)	49(43.8%)	63 (56.3%)	
Present	84 (42.9%)	28(33.3%)	56 (66.7%)	
**Comorbidities**				0.746
Absent	140(71.4%)	54 (38.6%)	86 (61.4%)	
Present	56(28.6%)	23 (41.4%)	33 (58.9%)	
**Hemoglobin**				0.465
<143.5 g/L	98 (50.0%)	36 (36.7%)	62 (63.3%)	
≥ 143.5 g/L	98 (50.0%)	41 (41.8%)	57 (58.2%)	
**Tumor differentiation**				0.869
Highly	96 (49.0%)	38 (39.6%)	58 (60.4%)	
Moderately	82 (41.8%)	31 (37.8%)	51 (62.2%)	
Poorly	18 (9.2%)	8 (44.4%)	10(55.6%)	
**Position**				0.933
Glottic	49 (25.0%)	19 (38.8%)	30(61.2%)	
Non-Glottic	147 (75.0%)	58 (39.5%)	89 (60.5%)	
**T classification**				0.002
1	16 (8.2%)	12 (75.0%)	41 (25.0%)	
2	180 (91.8%)	65 (36.1%)	115 (63.9%)	
**N classification**				0.007
0	177 (90.3%)	75 (42.4%)	102 (57.6%)	
1	19 (9.7%)	2 (10.5%)	17 (89.5%)	
**Clinical stage**				0.001
I	16 (8.2%)	12 (75.0%)	4 (25.0%)	
II	161 (82.1%)	63 (39.1%)	98 (60.9%)	
III	19 (9.7%)	2 (10.5%)	17 (89.5%)	
**Treatment method**				0.418
Surgery	160 (81.6%)	65 (40.6%)	95 (59.4%)	
Comprehensive Treatment	36 (18.4%)	12 (33.3%)	24 (66.7%)	
**HPV status**				0.989
Negative	163 (83.2%)	64 (39.3%)	99 (60.7%)	
Positive	33 (16.8%)	13 (39.4%)	20 (60.6%)	
**PI3K mutation status**				0.992
Yes	5(1.5%)	3(60%)	2(40%)	
No	191(97.4%)	101(52.9%)	90(47.1%)	
**Progression**				0.019
No	129 (65.8%)	61 (47.3%)	68 (52.7%)	
Yes	67 (34.2%)	16 (23.9%)	51 (76.2%)	
**Live status**				<0.001
Live	129 (65.8%)	64 (49.6%)	65 (50.4%)	
Dead	67 (34.2%)	13 (19.4%)	54 (80.6%)	

Abbreviations: LSCC, laryngeal squamous cell carcinoma; HPV, human papillary virus; PTOV1, prostate tumor overexpressed-1; Non-Glottic, Supraglottic and Subglottic; Comprehensive Treatment, Surgery and chemotherapy or Surgery and radiotherapy.

### Association between PTOV1 expression and survival outcomes in LSCC

**Figure 4:**
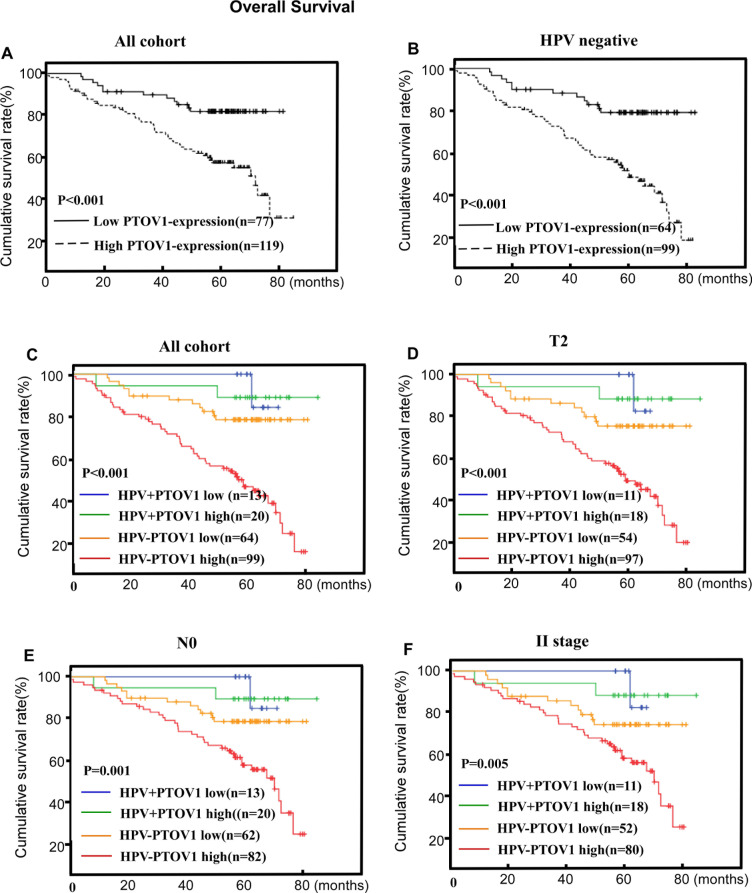
PTOV1 protein expression is associated with overall survival (OS) in the whole cohort **A.** and the HPV-negative subgroup **B.** in LSCC. PTOV1+HPV status is associated with OS in the T2 subgroup, the N negative subgroup and the stage II subgroup **C.**, **D.** and **E.**, respectively). P-values were calculated using the log-rank test.

**Figure 5:**
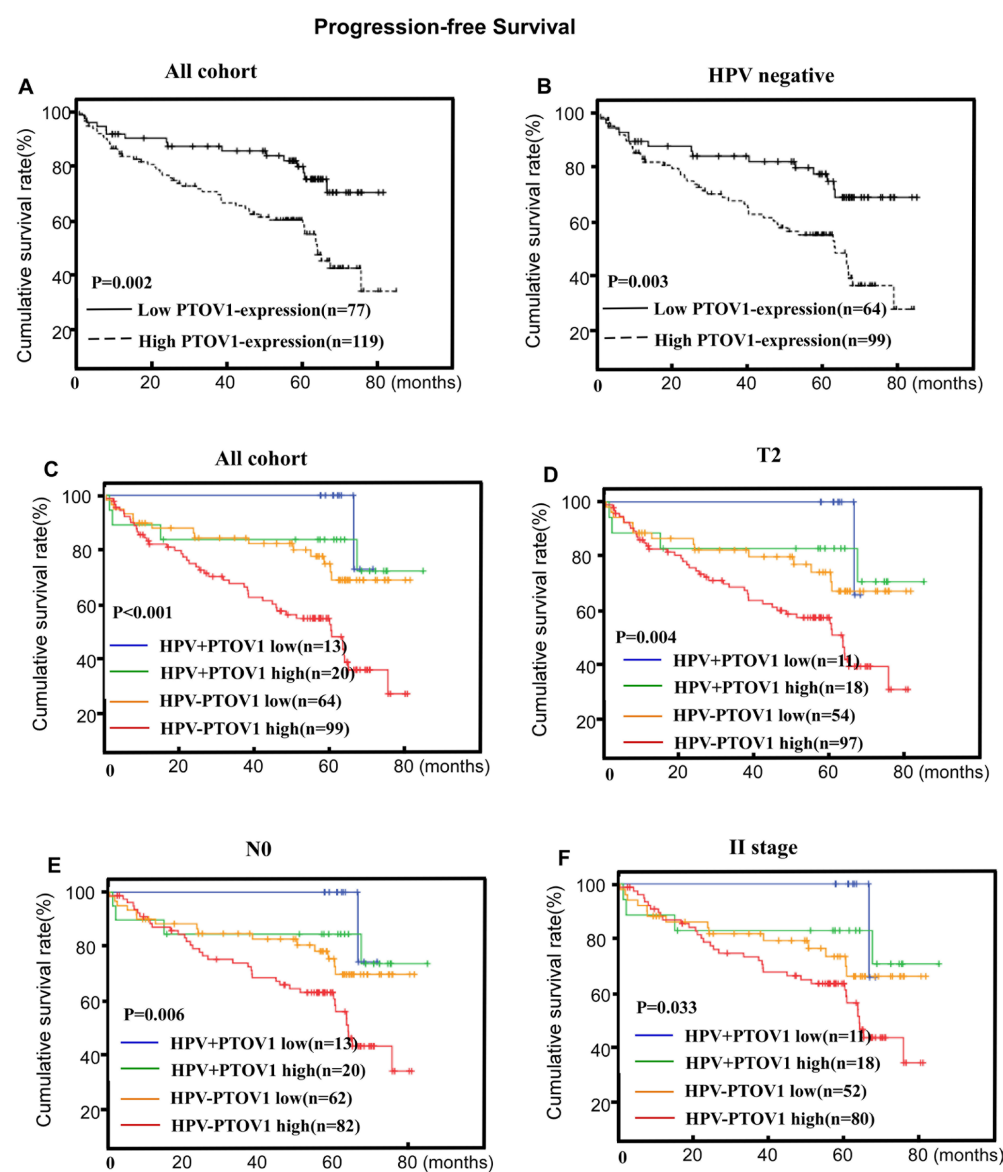
PTOV1 protein expression is associated with progression-free survival (PFS) in the whole cohort **A.** and the HPV-negative subgroup **B.** in LSCC. PTOV1+HPV status is associated with PFS in the T2 subgroup, the N negative subgroup and the stage II subgroup **C.**, **D.** and **E.**, respectively). P-values were calculated using the log-rank test.

Assessment of patients' survival by Kaplan-Meier analysis and the log-rank test revealed that high PTOV1 protein expression was associated with significantly poorer OS and PFS (*P* < 0.001 and *P* = 0.017, respectively Figures 4A, 5A). The cumulative 5-year OS rate and PFS rates for patients were 83.9 and 83.2% for lower PTOV1 expression subgroup, and 63.8% and 63.0% for higher PTOV1 expression subgroup, respectively.

Univariate Cox regression analysis showed that hemoglobin (*P* = 0.036), N stage (*P* < 0.001), treatment method (*P* = 0.006), HPV status (*P* = 0.004) and PTOV1 expression (*P* < 0.001), HPV/PTOV1 status (*P* < 0.001) were significant prognostic factors for OS. Likewise, N stage (*P* < 0.001), treatment method (*P* = 0.003), PTOV1 expression (*P =* 0.003) and HPV status (*P* = 0.011), HPV/PTOV1 status (*P* = 0.001) were significant prognostic factors for PFS in LSCC (Table 2).

Multivariate survival analysis was performed using the covariates that were statistically significant in the Univariate Cox regression analysis. As expected, PTOV1 (HR, 2.806; *P* = 0.001), hemoglobin (HR, 0.593; *P* = 0.038), N classification (HR, 3.960; *P* < 0.001) and positive HPV status (HR, 0.209; *P* = 0.009) were identified as independent prognostic factors for poor OS. Furthermore, PTOV1 (HR, 2.238; *P* = 0.005), N classification (HR, 2.138; *P* = 0.038) and HPV status (HR, 0.306; *P* = 0.012) were also independent prognostic factors for PFS (Table 3).

**Table 2:** Univariate Cox regression analysis of the association of various clinicopathological features with of overall survival (OS) and progression-free survival (PFS)

Feature	OS		PFS	
	HR (95% CI)	*P*	HR (95% CI)	*P*
Age (y) ≥45 *vs*. <45	1.154 (0.714-1.866)	0.558	1.007 (0.623-1.627)	0.978
Gender F VS M	0.539 (0.169-1.717)	0.296	0.518 (0.162-1.651)	0.266
Drinking status Present *vs*. Absent	0.860 (0.450-1.642)	0.647	1.245 (0.595-2.608)	0.561
Smoking status Present *vs*. Absent	1.321 (0.816-2.138)	0.258	1.006 (0.617-1.641)	0.982
Comorbidities Present *vs*. Absent	0.695 (0.391-1.235)	0.214	1.388 (0.838-2.299)	0.203
Hemoglobin ≥ 143.5 g/L *vs*. <143.5g/L	0.590 (0.361-0.966)	0.036	0.693 (0.427-1.124)	0.137
Pathological differentiation		0.114		0.175
Highly	Reference		Reference	
Moderately	1.671 (1.010-2.765)	0.046	1.458 (0.888-2.395)	0.136
Poorly	1.003 (0.387-2.598)	0.995	0.685 (0.241-1.952)	0.479
Site Non-glottic *vs*. Glottic	0.801 (0.471-1.363)	0.413	1.205 (0.668-2.172)	0.535
T stage T2 *vs*. T1	6.728 (0.933-48.518)	0.059	3.722 (0.911-15.216)	0.067
N stage N1 *vs*. N0	5.421 (3.006-9.777)	<0.001	3.445 (1.721-6.894)	<0.001
Treatment method Comprehensive *vs*. Surgery	0.463 (0.268-0.802)	0.006	0.443 (0.257-0.764)	0.003
HPV status Positive *vs*. negative	0.182 (0.057-0.580)	0.004	0.304 (0.122-0.759)	0.011
PTOV1 High *vs*. Low	3.065 (1.672-5.619)	<0.001	2.379 (1.356-4.174)	0.003
**HPV/PTOV1 status		<0.001		0.001
HPV+/PTOV1-	1.000		1.000	
HPV+/PTOV1+	1.220(0.110-13.479)	0.871	2.902(0.324-25.987)	0.341
HPV1-/PTOV1-	2.691(0.350-20.719)	0.342	3.906(0.515-29.591)	0.187
HPV-/PTOV1+	9.044(1.248-65.520)	0.029	9.245(1.273-67.122)	0.028

Abbreviations: LSCC, laryngeal Squamous cell carcinoma; H, highly differentiated; M, moderately differentiated; P, poorly differentiated; PTOV1, prostate tumor overexpressed-1 (PTOV1); HPV, human papillary virus; HR, hazards ratio.

** The clinicopathological features associated with the overall survival (OS) and progression-free survival (PFS) when combined the HPV status and PTOV1 expression level.

**Table 3:** Multivariate Cox regression analysis of the association of various clinicopathological features with overall survival (OS) and progression-free survival (PFS)

Features	OS		PFS	
	HR (95% CI)	*P*	HR (95% CI)	*P*
* stage N1 *vs*. N0	3.960 (2.163-7.250)	<0.001	2.138 (1.044-4.379)	0.038
HPV status Positive *vs*. negative	0.209 (0.065-0.673)	0.009	0.306 (0.122-0.772)	0.012
PTOV1 High *vs*. Low	2.806 (1.518-5.185)	0.001	2.238 (1.271-3.941)	0.005
Hemoglobin ≥ 143.5 g/L *vs*. <143.5g/L	0.593 (0.362-0.971)	0.038		
Treatment method Comprehensive *vs*. Surgery			1.987 (1.131-3.488)	0.017
**HPV-negative group PTOV1 High *vs*. Low	2.943 (1.557-5.563)	0.001	2.246 (1.250-4.034)	0.007
N stage N1 *vs*. N0	3.939 (2.151-7.212)	<0.001	2.139 (1.044-4.384)	0.038
Hemoglobin ≥ 143.5 g/L *vs*. <143.5g/L	0.595 (0.363-0.974)	0.039		
Treatment method Comprehensive *vs*. Surgery			1.985 (1.127-3.498)	0.018
***HPV/PTOV1 status		<0.001		0.004
HPV+/PTOV1-	1.000		1.000	
HPV+/PTOV1+	1.223 (0.111-13.521)	0.870	2.294 (0.253-20.813)	0.461
HPV1-/PTOV1-	2.472 (0.321-19.062)	0.385	3.334 (0.438-25.388)	0.245
HPV-/PTOV1+	7.278 (0.998-53.073)	0.050	7.449 (1.018-54.482)	0.048

Abbreviations: LSCC, laryngeal Squamous cell carcinoma; PTOV1, prostate tumor overexpressed-1 (PTOV1); HPV, human papillary virus; HR, hazards ratio.

* The clinicopathological features associated with the overall survival (OS) and progression-free survival (PFS);

** The subgroup analysis of the clinicopathological features associated with the overall survival (OS) and progression-free survival (PFS) in the HPV negative subgroup;

*** The clinicopathological features associated with the overall survival (OS) and progression-free survival (PFS) when combined the HPV status and PTOV1 expression level

### Combination of HPV with PTOV1 and outcomes

Within the HPV-negative group, patients with high PTOV1 expression had up to 2.943 fold increased risks of poorer OS and 2.246 fold increased risks of poorer PFS relative to those with low PTOV1 expression, after adjusting for clinicopathological factors (Table 3, Figures 4B, 5B). However, within the HPV-positive group, there was only a marginal adjusted increased risk (data not shown).

The combination of PTOV1 and HPV status appeared a superior measure of prognosis than HPV status alone for OS and PFS, after adjusting for clinicopathological factors with the significance still exists in the HPV negative subgroup. Additionally, the combined effects of the PTOV1 and the HPV status also shown in the Table 3 with the best prognostic outcomes were for patients in the HPV-positive/PTOV1 negative groups and the worst in those with HPV-negative/ PTOV1 -positive groups (Tables 3, Figures 4C, 5C). Additionally, a stratified analysis showed that the prognostic significance of the combination of PTOV1/HPV still existed in the T2 stage, N negative stage and clinical II stage subgroup (Figures 4D, 4E, 4F and 5D, 5E, 5F).

## DISCUSSION

The present study was firstly to demonstrate the association of increased PTOV1 expression with poor prognosis in LSCC patients. PTOV1 protein and mRNA levels were elevated in human LSCC, with a positive PTOV1 staining rate of 94.5% in the archived LSCC biopsies. Univariate and multivariate analyses showed that high PTOV1 expression is an independent predictor of poor OS and PFS. It strongly supported the hypothesis that this protein is involved in the progression of LSCC and may represent a biomarker for the identification of subsets of LSCC patients with a more aggressive form of the disease. Additionally, our data provided evidence that using the combination of PTOV1 and HPV status improves the prediction reliability of outcome, with the best outcome being observed in the HPV-positive/PTOV-negative subgroup, and the worst in the HPV-negative/PTOV-positive subgroup, which might warrant more aggressive treatment.

Ectopic expression of PTOV1 was first identified in prostate cancer [10]. Aberrant PTOV1 expression increased the number of cells that entered into the S phase of the cell cycle and increased tumor cell proliferation capacity; thus contributing to their biological behavior [12]. PTOV1 is a mitogenic protein that shuttles between the nucleus and cytoplasm to assist the translocation of the lipid-raft associated protein Flotillin-1 to the nucleus and the activation of mitogenic activity [16]. High levels of PTOV1 in prostatic tumors also correlated significantly with the Ki-67 proliferative index and its nuclear localization, suggesting a functional relationship between PTOV1 overexpression, proliferative status and nuclear localization [12]. Subsequently, the role of PTOV-1 overexpression in the proliferative status of tumor cells has been implicated in other neoplasms, such as breast, ovary and bladder cancers [10, 11, 17, 18].

Moreover, overexpression of PTOV1 in primary and metastatic tumors significantly increases the translation of active c-Jun and its nuclear localization [13]. C-jun is a major transcriptional factors of the AP-1 complex and regulate a variety of cellular fates, including proliferation, differentiation and apoptosis [19]. The role of AP1 complexes in promoting cancer cell invasion and metastasis is well established [20, 21]. C-Jun is a transcription factor of the phosphatidylinositol 3-Kinase/Akt/mTOR signaling pathway, which is essential in a variety of cellular processes, including proliferation, differentiation, cancer stem cells biology and tumor initiation and propagation [22]. Therefore, we assumed that PTOV1 is a positive regulator of LSCC tumor cell proliferation and progression that acts *via* the transcription factor c-Jun and its downstream genes. However, the exact mechanism needs further investigation.

LSCC is predominantly found in men, with a male to female ratio of approximately 6:1 [23]. This gender discrepancy may be ascribed to the enhanced activity and elevation of the androgen receptor (AR) in men, which promotes the growth of LSCC[24]. PTOV1 has been mapped to 19q13, which harbors a large number of genes whose expression are stimulated by androgens, and PTOV1 itself is activated by exposure to androgens [25]. Consistent with the AR-related theory above, our study revealed that males tend to be with the characteristics of high PTOV1-expressing, and might need more aggressive treatment strategy than females with the equivalent TNM stage.

In many other malignancies, multiple molecular markers, such as CD44, mircroRNAs expression are being used increasingly to predict prognosis and guide treatment [26, 27]. HPV-positive head and neck cancers have better prognosis than HPV-negative ones, especially given the general trend of earlier diagnoses of the more symptomatic laryngeal cancers [28]. However, the frequency of HPV detection in laryngeal lesions varies between 8% and 60%, leaving some uncertainty whether HPV-related laryngeal cancers represent a clinical subset unique from carcinomas with non-viral origins [29]. The early identification of PTOV1 expression in laryngeal lesions could also be a clinically independent biomarker to predict disease progression and survival. Our study has combined the PTOV1 and HPV status to evaluate the prognostic significance in LSCC. Our data provide evidence that using PTOV1 in conjunction with HPV improves the prediction of outcome in LSCC, especially for patients with HPV-negative/PTOV1-positive tumors, which might warrant more aggressive treatment. The significance of HPV-negative/PTOV-positive or HPV-positive/PTOV-negative was not observed, which may relate to tobacco or alcohol use of HPV-positive patients and the small sample size of the patients [30]. However, the underlying mechanism remains to be studied.

In conclusion, high PTOV1 expression was associated with advanced clinical stage and might stimulate LSCC development and progression. Additionally, we demonstrated that a combination of HPV and PTOV1 status provides more prognostic information for LSCC than HPV alone. Thus, PTOV1 might represent a useful molecular biomarker of poor prognosis in LSCC. Clinical diagnosis combined with the usage of these biomarkers may improve stratified prognosis estimation and help to identify high-risk LSCC patients who may benefit from more aggressive treatment. However, while this study offers some preliminary data on a role for PTOV-1 in laryngeal carcinoma, the underlying molecular mechanisms of PTOV-1 in LSCC development and progression require further investigation. The main limitation of the manuscript is the lack of the cell-functional experiment to further demonstrate the mechanism of the association of the high-expression of the PTOV1 and the poor prognosis in LSCC

## MATERIALS AND METHODS

### Patients and tissue specimens

This study was conducted on a total of 196 paraffin-embedded LSCC samples that were histologically and clinically diagnosed at Sun Yat-sen University Cancer Center between 2000 and 2009. Prior written patient consent was obtained and the Institutional Research Ethics Committee of Sun Yat-sen University Cancer Center approved the protocols. The clinical classifications of all the patients were classified or reclassified according to the seventh edition of the American Joint Committee on Cancer system. Clinical information for the samples is summarized in Table 1.

All patients received definitive surgery based on their clinical stage, including 14 cases of total laryngectomy, 163 cases of hemilaryngectomy, and 19 cases of CO_2_ laser endoscopic resection, all of which with selective or definitive neck dissection [31]. Ninety-six patients received postoperative radiotherapy according to institutional guidelines and no chemotherapy.

Median follow-up for overall survival (OS) and progression free survival (PFS) were 64.3 months and 62.3 months, respectively. The five-year OS rate and PFS rates were 64.3 months and 63.8. A total of 67/196 (34.2%) died and 67/196 (34.2%) patients experienced distant metastasis or recurrence during follow-up.

### HPV DNA detection and genotyping

An HPV-positive cancer was defined as one testing positive for HPV DNA and overexpressing p16 [3]. The existence and type of HPV DNA were detected using an E6-based multiplex tandem-PCR assay (MT-PCR) which was performed on four to six 4-5μm sections of tumor using the method described by Stanley [32]. This assay could simultaneously identify 21 HPV types (16, 18, 31, 33, 35, 39, 45, 51, 52, 56, 58, 59, 66, 68, 70, 73, 82, 53, 6, 11 and 26). Genomic DNA was extracted with a QIAamp DNA FFPE Tissue Kit (Qiagen Ltd, Hilden, Germany). All primer pairs were incorporated into two PCR mixes and amplified for 20 cycles in a GeneAmp 2700 thermocycler (Applied Biosystems, Australia). Triplex TaqMan real-time PCR assays with probes having FAM, VIC or Cy5 labels, using the products from these mixes were performed for 40 cycles of amplification in a RotorGene 6000 real-time thermocycler (Corbett Research, NSW, Australia). To avoid cross contamination, the paraffin sections were cut with stringent precautions and water blanks were substituted after every fifth tube to detect cross contamination.

### PIK3CA/H-RAS gene amplification and sequencing

Genomic DNA was evaluated for the presence of H-RAS, PIK3CA mutations using PCR amplification and direct sequencing. H-RAS and PIK3CA were amplified using the following premiers: H-RAS exon 3 forward 5′-AGAGGCTGGCTGTGTGAACT-3′ and reverse 5′-TGGTGTTGTTGATGGCAAAC-3′. PI3K exons 9 forward 5′-CTGTGAATCCAGAGGGGAAA-3′ and reverse 5′-TTTAGCACTTACCTGTGACTCCA-3′; PIK3CA exon 20 forward 5′-CTCAATGATGCTTGGCTCTG-3′ and reverse 5′-TTTTCAGTTCAATGCATGCTG-3′. PCR was carried out in 20 μl of reaction mixture containing 60~200 ng of genomic DNA, 0.4 μmol/l of each primer, dNTP mixture 1.6 μl, 10×buffer 2μl, and TaKaRa Taq 0.5 U. After an initial denaturation step at 95°C for 5 min, 30 seconds at 95°C, 30 seconds at 48°C, 30 seconds at 72°C 35 cycle; then, 10 min at 72°C and 4°C on hold. PCR products were run on 1.2% agarose gels to verify the adequacy of the amplification.

The PCR products were purified using the magnetic bead method. Direct sequencing was performed with an ABI 3730xl DNA Analyzer using the BigDye^®^ Terminator v3.1 DNA Analyzer Sequencing Standards Kit (Applied Biosystems, Warrington, UK) according to the manufacturer's instructions. Any samples suspected of genetic alteration were subsequently sequenced in the reverse direction using the reverse primer. The purification and sequencing of the PCR products were performed in Life Technologies Corporation in China.

### RNA extraction and quantitative real-time PCR (RT-PCR)

Total RNA was extracted from tissues using the TRIzol reagent (Invitrogen), according to the manufacturer's instructions, treated with RNase-free DNase, and 2.0 μg of total RNA from each sample was subjected to cDNA synthesis using random hexamers. For PCR amplification of PTOV1 cDNA, an initial amplification using PTOV1-specific primers was performed with a denaturation step at 95°C for 10 min; followed by 30 cycles of denaturation at 95°C for 60 s, primer annealing at 55°C for 30 s and extension at 72°C for 30 s. Upon completion of the cycling steps, a final extension was carried out at 72°C for 5 min before the reaction was stopped and stored at 4°C. PCR primers *were designed using the Primer Express v 2.0 software (Applied* Biosystems) to determine the fold increases in PTOV1 mRNA expression in the tumor specimens relative to non-cancerous tissues. The sequences of the PCR primers were: PTOV1 forward 5ʹ- CGAGTACAGGAGCATGAGCA-3ʹ and Reverse: 5ʹ-CTTCACCAACAGAGACTGCG-3ʹ; GAPDH Forward: 5ʹ-GACTCATGACCACGTCCATGC-3ʹ and Reverse: 5ʹ-AGAGGC

AGGGATGATGTTCTG-3ʹ. PTOV1 expression data was normalized to *GAPDH* expression; all experiments were performed in triplicate.

### Western blotting

Fresh tissue samples were ground to a powder in liquid nitrogen and lysed with SDS-PAGE sample buffer (62.5 mmol/L Tris-HCl pH 6.8, 2% SDS, 10% glycerol, and 5% 2-mercaptoethanol). Protein concentrations were determined using the Bradford assay (Bio-Rad Laboratories, Hercules, CA, USA). Equal amounts of protein samples (30 μg) were separated on 10.5% SDS polyacrylamide gels and transferred to polyvinylidene difluoride membranes (Immobilon P, Millipore, Bedford, MA). Membranes were blocked with 5% fat-free milk in Tris-buffered saline containing 0.1% Tween-20 (TBST) for 1 h at room temperature, incubated with anti-PTOV1 rabbit polyclonal antibody (1:100; Sigma, HPA051812) overnight at 4°C, and then with horseradish peroxidase-conjugated goat anti-rabbit IgG (1:1000, Santa Cruz Biotechnology, SC-2004). The membranes were probed with anti-α-tubulin mouse monoclonal antibody (1:1000, Sigma, T5168) as a loading control. PTOV1 expression was detected using enhanced chemiluminescence system (ECL) prime western blotting detection reagent (Amersham), according to the manufacturer's instructions. The loading control was α-tubulin.

### Immunohistochemistry (IHC)

IHC was performed to measure PTOV1 protein expression in the 196 human LSCC tissues. In brief, 4 μm-thick paraffin-embedded sections were baked at 60°C for 2 h, followed by deparaffinized with xylenes and rehydration, before being microwaved in EDTA antigen retrieval buffer. The sections were then treated with 3% hydrogen peroxide in methanol to quench endogenous peroxidase activity, incubated with 1% bovine serum albumin to block non-specific binding and incubated with an anti-p16 antibody (JC8, Santa Cruz, CA, 1/100) or an anti-PTOV1 rabbit polyclonal antibody (1:50, Sigma, HPA051812) at 4°C overnight. For negative controls, the primary antibody was replaced by normal goat serum. After washing, the tissue sections were treated with biotinylated anti-rabbit secondary antibody (Abcam) and incubated with streptavidin horseradish peroxidase complex (Abcam). The tissue sections were immersed in 3-amino-9-ethyl carbazole, counterstained with 10% Mayer's hematoxylin, dehydrated and mounted in Crystal Mount.

Two independent observers, who were blinded to the histopathological features and patient data of the samples, scored the degree of immunostaining of the sections. The p16 antibody stained both the nucleus and cytoplasm of tumor cells. Staining was almost invariably strong and diffuse, and essentially all or none. Weak focal staining was recorded as negative. The proportion of PTOV1-expressing cells varied from 0% to 100% and the staining intensity varied from undetectable to strong. The intensity of staining was graded as 0 (no staining), 1 (weak, light yellow), 2 (moderate, yellowish brown) and 3 (strong, brown). The proportion of positive tumor cells was recorded as: 0 (no positive tumor cells), 1 (1%-10%), 2 (11%-35%), 3 (36%-70%) and 4 (> 70%). The staining index was calculated as the product of the proportion of positive cells and the staining intensity score for each section (to obtain values of 0, 1, 2, 3, 4, 6, 9 or 12). The cut-off values for PTOV1 expression were chosen based on a measure of heterogeneity, using the log-rank test with respect to OS and PFS. A staining index score ≥ 6 was used to classify tumors with high expression and a score of ≤ 4 indicated low PTOV1 expression.

The method of mean optical density (MOD) was used to determine the immunostaining intensity of each tested specimen and was performed as previously reported [33]. tive pixels in the whole tissue.

A negative control with each batch of staining was used for background subtraction in the quantitative analysis. The MOD data were statistically analyzed using *t*-test to compare the average MOD difference between different group of tissues, *P* < 0.05 was considered significant.

### Statistical analyses

All statistical analyses were carried out using the SPSS 19.0 statistical software packages. Pearson's χ^2^ and Fisher's exact tests were used to analyze the associations between PTOV1 expression and clinicopathological features. Bivariate correlations between the study variables were calculated using Spearman's rank correlation coefficients. OS was defined as the time from start of treatment to death or end of follow-up and PFS was the time from start of treatment to onset of recurrence, distant metastasis or end of follow-up, as diagnosed by clinical assessment or MRI imaging. Survival curves were plotted using the Kaplan Meier method and compared with the log-rank test. Survival data were evaluated using univariate and multivariate Cox regression analyses. A two-sided probability value < 0.05 was considered statistically significant.
